# Innovations in surgical training: exploring the role of artificial intelligence and large language models (LLM)

**DOI:** 10.1590/0100-6991e-20233605-en

**Published:** 2023-08-14

**Authors:** JULIAN VARAS, BRANDON VALENCIA CORONEL, IGNACIO VILLAGRÁN, GABRIEL ESCALONA, ROCIO HERNANDEZ, GREGORY SCHUIT, VALENTINA DURÁN, ANTONIA LAGOS-VILLASECA, CRISTIAN JARRY, ANDRES NEYEM, PABLO ACHURRA

**Affiliations:** 1 - Pontificia Universidad Católica de Chile, Experimental Surgery and Simulation Center, Department of Digestive Surgery - Santiago - Región Metropolitana - Chile; 2 - Pontificia Universidad Católica de Chile, Carrera de Kinesiología, Departamento de Ciencias de la Salud, Facultad de Medicina - Santiago - Región Metropolitana - Chile; 3 - Pontificia Universidad Católica de Chile, Computer Science Department, School of Engineering - Santiago - Región Metropolitana - Chile; 4 - Pontificia Universidad Católica de Chile, Department of Otolaryngology - Santiago - Región Metropolitana - Chile

**Keywords:** Artificial Intelligence, Education, Medical, Learning Curve, Inteligência Artificial, Educação Médica, Curva de Aprendizado

## Abstract

The landscape of surgical training is rapidly evolving with the advent of artificial intelligence (AI) and its integration into education and simulation. This manuscript aims to explore the potential applications and benefits of AI-assisted surgical training, particularly the use of large language models (LLMs), in enhancing communication, personalizing feedback, and promoting skill development. We discuss the advancements in simulation-based training, AI-driven assessment tools, video-based assessment systems, virtual reality (VR) and augmented reality (AR) platforms, and the potential role of LLMs in the transcription, translation, and summarization of feedback. Despite the promising opportunities presented by AI integration, several challenges must be addressed, including accuracy and reliability, ethical and privacy concerns, bias in AI models, integration with existing training systems, and training and adoption of AI-assisted tools. By proactively addressing these challenges and harnessing the potential of AI, the future of surgical training may be reshaped to provide a more comprehensive, safe, and effective learning experience for trainees, ultimately leading to better patient outcomes. .

## RESUMO

O cenário do treinamento cirúrgico está evoluindo rapidamente com o surgimento da inteligência artificial (IA) e sua integração na educação e simulação. Este artigo explora as aplicações e benefícios potenciais do treinamento cirúrgico assistido por IA, em particular o uso de modelos de linguagem avançados (MLAs), para aprimorar a comunicação, personalizar o feedback e promover o desenvolvimento de habilidades. Discutimos os avanços no treinamento baseado em simulação, ferramentas de avaliação impulsionadas por IA, sistemas de avaliação baseados em vídeo, plataformas de realidade virtual (RV) e realidade aumentada (RA), e o papel potencial dos MLAs na transcrição, tradução e resumo do feedback. Apesar das oportunidades promissoras apresentadas pela integração da IA, vários desafios devem ser abordados, incluindo precisão e confiabilidade, preocupações éticas e de privacidade, viés nos modelos de IA, integração com os sistemas de treinamento existentes, e treinamento e adoção de ferramentas assistidas por IA. Ao abordar proativamente esses desafios e aproveitar o potencial da IA, o futuro do treinamento cirúrgico pode ser remodelado para proporcionar uma experiência de aprendizado mais abrangente, segura e eficaz para os aprendizes, resultando em melhores resultados para os pacientes.

## INTRODUCTION

Surgical training has traditionally followed the apprenticeship model, in which trainees learn from mentors by observing and assisting experienced surgeons in the operating room^1^. This approach, though valuable, is not without limitations. As surgical procedures have become increasingly complex and specialized, the need for more structured and standardized training methods has grown^2^ ^,^ ^3^. Concerns about patient safety and the need to objectively evaluate trainees’ performance have prompted the search for innovative and reliable training modalities^4^ ^,^ ^5^. The rise of Artificial Intelligence assisted education and simulation has become a relevant component in surgical training, enabling a more comprehensive, safe, and efficient learning experience for trainees^6^ ^-^ ^10^.

Artificial intelligence (AI) is a multidisciplinary field of computer science focused on creating intelligent agents capable of performing tasks that typically require human-like cognition^11^. AI systems employ techniques such as machine learning, deep learning, natural language processing, computer vision, and expert systems to perceive, reason, learn, and adapt to new information^11^. Machine learning enables computers to learn from data and make predictions or decisions without explicit programming, while deep learning uses artificial neural networks for sophisticated pattern recognition^12^. Natural language processing allows AI systems to understand and generate human-like text or speech, and computer vision deals with the analysis and interpretation of visual information^13^. Expert systems involve rule-based decision-making based on predefined knowledge bases^14^. By integrating these diverse AI methodologies, researchers can develop intelligent agents to tackle complex problems and transform various aspects of human life, including surgical training and education.

While there have been significant advancements in AI-assisted surgical training, the specific use of LLMs in facilitating communication and information processing within surgical training environments has been relatively understudied. Effective communication is a crucial aspect of surgical training, and leveraging LLMs to transcribe and translate feedback inputs from trainers to trainees can greatly improve the learning experience and outcomes. 

The paper aims to highlight the potential of LLMs in addressing this communication gap and explores their applications in transcription, translation, summarization of feedback data, and providing real-time corrections and recommendations to instructors. By addressing this gap, the paper contributes to the understanding of how LLMs can enhance surgical education and bridge the communication divide between trainers and trainees.

### AI-driven assessment and feedback

Simulation-based training, in particular, has proven to be a powerful adjunct to a traditional apprenticeship. It offers a controlled and risk-free environment where trainees can practice their skills before applying them in the operating room^15^ ^,^ ^16^. AI has significantly expanded the potential of simulation-based training in various ways^17^. One of the key advancements is the ability to provide personalized feedback to surgical trainees^6^. By analyzing performance metrics and identifying areas of improvement, AI-powered systems can tailor feedback to individual subjects, addressing their unique strengths and weaknesses^18^. This customized approach to feedback not only enhances the learning process but also enables trainees to focus on specific areas that require further development.

In addition to personalized feedback, AI is revolutionizing the assessment processes in surgical training. Traditional methods often rely on subjective evaluations by human observers, which can be prone to bias and inconsistency^15^ ^,^ ^19^. AI-driven assessment tools, on the other hand, can objectively measure surgical performance by analyzing various data sources, such as motion tracking, force measurements, historic feedback inputs, and video recordings^20^ ^,^ ^21^. Some of these tools can provide real-time feedback and generate standardized performance scores, thus ensuring a more consistent and reliable evaluation of trainee progress^7^ ^,^ ^18^.

Moreover, AI has facilitated the rise of video-based surgical assessment systems that leverage computer vision and machine learning techniques to analyze surgical videos and extract valuable insights^7^. These systems offer data-driven feedback and objective evaluations, enabling trainees and experienced surgeons to identify areas for improvement and learn from best practices^22^ ^,^ ^23^ (Table 1). 

**Table 1:** AI-enhanced Video-Based Surgical Assessment and Training Systems.

System	Description
Theator	A video-based surgical assessment platform that uses AI and computer vision technology to analyze surgical videos. Provides data-driven insights by annotating critical moments and key steps during procedures, enabling surgeons and trainees to review their performance, identify areas for improvement, and learn from best practices. Compares individual surgeon performance against established benchmarks, allowing for objective assessments and personalized feedback.
CSATS (Johnson & Johnson)	A surgical video review and assessment platform that leverages AI and data analytics to evaluate surgical performance. Provides objective feedback and recommendations based on expert peer reviews and data-driven insights. Offers educational content, such as lectures and case studies, to help surgeons improve their skills and techniques. CSATS encompasses a broad range of surgical specialties, including general surgery, orthopedics, and urology.
System	Description
Surgical AI	A cloud-based platform that uses AI to analyze surgical videos and extract relevant data for performance assessment and improvement. Offers real-time feedback, detailed performance reports, and recommendations for best practices. Supports a wide range of surgical procedures and specialties. It can be integrated with existing video recording systems in operating rooms, making it easily accessible for surgical teams.
Touch Surgery	A mobile app that provides AI-driven interactive surgical simulations and can also be used to analyze surgical videos for performance assessment and improvement. Offers real-time feedback and performance tracking. Covers a wide range of surgical procedures across various specialties. Allows trainees to practice their skills, test their knowledge, and track their progress over time.

### AI-assisted predicting of performance and skill development

Another promising application of AI in surgical training is the prediction of trainees’ surgical performance and skill development. By utilizing machine learning algorithms, AI systems can analyze historical performance data and identify patterns that can be used to predict future performance and technique progression^18^. This predictive capability can help trainers identify trainees in need of additional support, as well as optimize training curricula based on individual learning trajectories. Now that learning is moving from fixed training curriculums to competency-based curriculums, AI provides a more objective and less time-consuming assessment of trainees’ learning curves.

### AI and immersive technologies

AI is playing a major role in the advancement of virtual reality (VR) and augmented reality (AR) systems, especially in the field of surgical training. These immersive technologies offer realistic and interactive training scenarios that can closely resemble real-life surgical procedures^24^ ^-^ ^26^. AI algorithms can be employed to generate complex, dynamic environments and unique patient-based anatomical models, allowing trainees to practice various surgical techniques and approaches in a controlled and safe setting. Additionally, AI can facilitate real-time adaptation of the VR and AR scenarios based on the trainee’s performance, ensuring a more engaging and effective learning experience^25^. Video-based feedback and evaluation are scaling fast with the help of AI. Video-recorded practices are now automatically assessed by AI algorithms, and “copilot-like” recommendations for instructors when providing feedback to trainees are now possible^17^ ^,^ ^25^ (Table 2).

**Table 2:** Comparison of some AI-Driven Surgical Simulation Training Systems.

System	Description
Touch Surgery	Provides interactive surgical simulations using AI algorithms to analyze users' performance and provide real-time feedback. Covers a wide range of surgical procedures across various specialties, allowing trainees to practice their skills, test their knowledge, and track their progress over time.
Osso VR	A virtual reality (VR) surgical training platform that employs AI to deliver realistic and immersive simulations for various surgical procedures. Offers real-time performance metrics, personalized feedback, and objective assessments, enabling trainees to hone their skills in a controlled and risk-free environment.
Mimic Technologies	Offers the Da Vinci Skills Simulator, a simulation platform designed for robotic surgery training. Uses AI-powered algorithms to analyze users' performance and provide objective feedback based on multiple performance metrics and focusing on different aspects of robotic surgery, including dexterity, precision, and efficiency.
Surgical Science	Provides simulation training solutions, including the LapSim and EndoSim systems, designed for laparoscopic and endoscopic surgery training, respectively. Utilizes AI algorithms to offer real-time feedback, objective assessments, and performance tracking, helping trainees develop their skills and competencies in a safe and controlled setting.
Lapp Simulation Training	Uses AI to understand when trainees commit mistakes during training and recommends which type of feedback inputs instructors should provide asynchronously. Aims to enhance the learning process by offering targeted feedback based on individual-trainee performance and needs.

### Large Language Models in surgical education

Large language models (LLMs), a subset of generative AI models, have gained significant attention in recent years due to their ability to generate human-like text based on input data. These models are trained on vast amounts of text from diverse sources, enabling them to understand and generate contextually appropriate responses in various languages and domains^27^. Examples of LLMs include OpenAI’s GPT-3 and the recently launched GPT-4. Despite the numerous advances in AI-assisted surgical training, there is still a notable gap in the literature regarding applying large language models (LLMs) to surgical education, for instance, in facilitating the transcription and translation of feedback inputs from trainers to trainees in simulated and video-based assessment environments. This gap is significant because effective communication is a critical aspect of surgical training, and addressing it could lead to considerable improvements in the learning experience and outcomes for trainees^28^ ^,^ ^29^.

LLMs have demonstrated remarkable capabilities in understanding and generating human-like text, making them well-suited for tasks related to communication and information processing^27^. In surgical training, LLMs could be employed to automatically transcribe spoken feedback provided by trainers during simulations or video-based assessments. This transcription could be used to create a written record of the feedback, allowing trainees to review and reflect on the suggestions and guidance received.

LLMs could also be utilized to translate feedback into different languages even in real time, thus overcoming potential language barriers between trainers and trainees. With surgical training programs increasingly attracting students from different countries, language gaps can be a significant challenge to effective communication. By leveraging LLMs to provide real-time or asynchronous translation, trainers and trainees could communicate more effectively, ensuring that valuable feedback is accurately conveyed and understood^30^. 

Another potential application of LLMs in surgical training is the extraction and summarization of key insights from large volumes of feedback data^31^. By analyzing feedback from multiple trainers or across numerous training instances, LLMs could identify recurring themes or patterns and provide students with concise, actionable summaries. This approach could help trainees to prioritize their learning objectives and focus on the most critical areas for improvement.

Similarly, LLM systems have the potential to improve instructors’ performance by providing real-time corrections and feedback, helping them meet minimum parameters and gradually improving their communication skills with trainees. The best clinician expert is not always the best at teaching and communicating learning experiences. However, semantic feedback from an automatic artificial intelligence system may help the sender notice areas for improvement in their teaching abilities.

### LLMs challenges in surgical training

Integrating large language models (LLMs) into surgical training comes with various challenges, including ensuring the accuracy and reliability of LLM-generated transcriptions and translations, addressing ethical and privacy concerns, and handling potential biases in the AI models. Below, we explore these challenges in greater detail:

- Accuracy and reliability: LLM-generated transcriptions and translations must be accurate and reliable to avoid misunderstandings or misinterpretations that could negatively impact the learning process. Inaccurate transcriptions might cause trainees to miss crucial feedback or make incorrect decisions based on flawed information. Ensuring the quality of LLM-generated content requires continuous monitoring, validation, and improvement of the models to minimize errors and maximize the value of the AI-generated outputs.
- Ethical and privacy concerns: The use of AI-generated feedback in surgical training may raise ethical and privacy concerns, particularly when sensitive patient information is involved. Ensuring compliance with data protection regulations, such as the Health Insurance Portability and Accountability Act (HIPAA) in the United States or the General Data Protection Regulation (GDPR) in the European Union, is essential. Implementing data anonymization techniques, secure storage and transmission protocols, and access controls can help protect sensitive information and maintain patient confidentiality.
- Bias in AI models: AI algorithms, including LLMs, can inadvertently propagate biases present in the training data. These biases may manifest in the form of gender, racial, or cultural stereotypes, potentially leading to unfair or discriminatory feedback. To address this issue, it is essential to develop transparent and fair AI models by using diverse and representative training data, applying bias-mitigation techniques, and rigorously testing the algorithms to identify and correct potential biases.
- Integration with existing training systems: Integrating LLMs into surgical training may require substantial modifications to existing training platforms and workflows. This process can be complex and time-consuming, requiring close collaboration between developers, trainers, and trainees to ensure seamless integration and minimize disruptions to the training process.
- Training and adoption: The successful implementation of LLMs in surgical training requires trainers, trainees, and their institutions to adapt to new technology and its workflows. This may involve providing additional training to familiarize users with the AI tools and addressing any resistance to change. Ensuring user buy-in is crucial for the effective adoption and use of LLMs in surgical training environments.
- Cost and Talent: Implementing artificial intelligence in surgical training necessitates substantial financial investment and specialized human expertise. To embark on an AI project may entail considerable expenses, making it challenging to conduct a proper cost-benefit assessment initially. Nonetheless, major companies have significantly invested in developing algorithms that can be tailored to specific domains, thereby enhancing their potential. Although the initial costs of these services may be high, as is typically the case with technological evolution, these are expected to decrease over time. At present, the cost and infrastructure needed to incorporate AI into surgical training continue to pose significant challenges.

Addressing these challenges is essential to harness the full potential of LLMs in surgical training and ensure their successful integration into training programs and educational institutions. By proactively identifying and addressing these challenges, it is possible to develop effective AI-assisted surgical training solutions that may improve communication, enhance the learning experience, and ultimately contribute to better patient outcomes.

## DISCUSSION AND CONCLUSION

The integration of AI into our society and daily activities has begun progressively. As with other technologies, the surgical field has quickly adopted some of its benefits, and the complexity of surgical training has made AI an interesting venture. We have presented two main ways in which AI-related technologies are becoming part of the teaching experience.

Firstly, AI-enhanced assessment methods are being used. Skill acquisition involves a trainer, a trainee, assessment tools, feedback delivery, and a training instance. We have described some of the available tools or apps that can optimize the integration of these five aspects of surgical training. Video-based platforms are showing promising results, allowing not only AI-driven assessment (using various measurements) but also providing relevant feedback based on previous and contemporary data. On the other hand, LLM-based technologies address communication issues that are critical for the learning process. Even when precise metrics and formidable progress tracking are assured, feedback and knowledge must be delivered correctly to make significant improvements in the trainee. LLMs can help ensure that concepts are conveyed effectively, suggest feedback to trainers, and help overcome language barriers by translating in real-time.

Despite the numerous benefits, implementing AI-based technologies and especially LLMs in surgical training poses significant challenges that can be approached from two perspectives. Firstly, from a technological standpoint, integrating AI-based tools and platforms into daily surgical education requires new “know-how,” and the integration of novel and “old” systems may be disruptive, difficult, and expensive. Careful and gradual integration may reduce resistance to these innovative technologies, especially in more experienced and traditional institutions. Secondly, from an educational perspective, concerns may arise regarding the reliability of “automatic” assessments, feedback, and inputs. Regarding this topic, it is important to understand that AI is a probabilistic model used to analyze data, employing high-powered computer technology to organize information from many sources. Currently, AI does not create new information in the strict sense of the word, so many of its limitations and mistakes are attributable due to the low quality or insufficiency of its sources. Future research and development should aim to identify the best sources and provide filters to prevent inaccurate information from affecting the algorithm’s outcomes. It is our responsibility to develop and apply safety measures to track interactions and identify any issues that may lead to misunderstandings or negatively impact the learning experience. Ensuring data protection, reducing bias, and testing the real educational impact of AI-driven tools are other significant challenges that we will face in adopting these technologies. 

In addition to the technical considerations, it is essential to highlight the ethical and legal challenges associated with the use of AI and LLMs in surgical practice.

From an ethical perspective, AI and LLMs in surgery raise several concerns. The first one pertains to patient autonomy and informed consent. It’s crucial to ensure that patients thoroughly understand the nature and extent of AI and LLM involvement in their treatment. Given the complexity of these technologies, conveying this information in a way that a layperson can understand is challenging.

The second ethical concern relates to accountability. In instances where AI or an LLM might make a mistake or provide suboptimal advice, it’s not immediately clear who would be held responsible - the developers, the medical practitioners relying on the technology, or the institutions adopting it. This ambiguity could lead to ethical dilemmas in practice.

Thirdly, there is the question of bias and fairness. AI and LLMs are trained on vast datasets and can inadvertently perpetuate existing biases in these data, leading to unequal treatment outcomes. For instance, if the training data are skewed towards a particular demographic, the AI might be less effective when applied to patients from underrepresented demographics.

Turning to the legal challenges, the application of AI and LLMs in surgical practice sits at the intersection of several legal domains, including data protection, intellectual property rights, and medical malpractice. AI’s ability to process large amounts of patient data raises questions about data privacy and security. Furthermore, who owns the intellectual property rights to AI and LLM-assisted surgical techniques or inventions? This is an area of law that is still evolving and has yet to catch up with the rapid advancements in AI technology.

In the event of an adverse outcome, the legal liability is also a grey area. If an error occurs due to the advice or assistance of an AI or LLM, traditional medical malpractice doctrines may not apply. The use of AI could blur the lines of responsibility, making it difficult to determine who is at fault.

Although the potential benefits of AI and LLMs in surgical training are significant, it is important to recognize and discuss inherent limitations of these technologies. First, AI and LLMs are dependent on the data they are trained on. If the data is incomplete, biased, or not representative of the range of cases a surgeon might encounter, the training and advice given may be suboptimal. Additionally, these technologies may lack the nuanced understanding of a human trainer, who can interpret complex visual cues and adapt to the trainee’s individual learning style in a way that AI and LLMs currently cannot.

Another limitation is the risk of over-reliance on technology. Surgical trainees might rely too heavily on AI and LLMs, which could impair the development of their independent decision-making skills. Furthermore, the high costs associated with the development and implementation of AI and LLMs may make these technologies inaccessible to some institutions, exacerbating existing inequities in surgical training.

Looking to the future, several exciting research directions are emerging. We need rigorous studies evaluating the impact of AI and LLMs on long-term surgical performance and patient outcomes, which can help validate the effectiveness of these technologies. Novel approaches could also be explored, such as integrating AI and LLMs with virtual or augmented reality to create immersive surgical training experiences. Furthermore, research should address the aforementioned limitations. For example, studies could investigate how to effectively combine AI and human mentorship in surgical training, or how to make these technologies more accessible to a broader range of institutions.

As a conclusion, as technology advances and AI becomes more integrated into the medical field, it is crucial to address the various challenges and limitations associated with AI technology while exploring new applications and opportunities in surgical education. By embracing the transformative power of AI, the future of surgical training can be reshaped to provide a more comprehensive, safe, and effective learning experience for trainees.
